# Stercoral colitis complicated with ischemic colitis: a double-edge sword

**DOI:** 10.1186/s12876-017-0686-6

**Published:** 2017-11-28

**Authors:** Maliha Naseer, Jenil Gandhi, Noor Chams, Zain Kulairi

**Affiliations:** Department of Internal Medicine, Wayne State University-School of Medicine, 1101 W University Dr. 2-South, Rochester, MI 48307 USA

**Keywords:** Stercoral colitis, Ischemic colitis, Chronic constipation, Lactic acidosis

## Abstract

**Background:**

Stercoral colitis is a rare inflammatory process involving the colonic wall secondary to fecal impaction with high morbidity and mortality; especially if complicated with ischemic colitis, stercoral ulcer formation and subsequent perforation. There are several case reports published on abdominal perforation resulting from stercoral colitis. However, stercoral colitis complicated by ischemic colitis is rare. The purpose of this case report is to describe the potential challenges in the diagnosis and management of stercoral colitis with ischemic colitis.

**Case presentation:**

An 87 years old male with history of chronic constipation presents with severe abdominal pain to the emergency department. The patient was hemodynamically stable. On physical examination, the abdomen was mildly distended with moderate tenderness. Lab work was significant for leukocytosis and lactic acidosis. Abdominal CT scan revealed large amount of retained stool in the colon, bowel wall thickening and infiltration of peri-colonic fat, which were suggestive for stercoral colitis. Patient was started on IV fluids and antibiotics. He was given an enema, followed by laxative and manual disimpaction of stool. Colonoscopy was performed and biopsies were obtained. Tissue biopsy was significant for focal active colitis with regenerative glandular changes and neural hyperplasia.

**Conclusion:**

Elevated lactic acid level secondary to ischemia of the bowel wall with CT scan findings aid in establishing the diagnosis of stercoral colitis complicated with ischemic colitis. Urgent treatment with laxatives and fecal disimpaction is indicated to prevent perforation and peritonitis.

## Background

Stercoral colitis is a rare yet serious inflammatory condition that carries a high morbidity and mortality, especially if complicated with intestinal perforation and ischemic colitis. Chronic constipation has been described as a risk factor for stercoral colitis and colonic ischemia [1, 2]. Patients can be asymptomatic or can present with vague symptoms that could be confused with diverticulitis, a more common condition in elderly. The mechanism by which stercoral colitis leads to ischemia and colonic wall perforation is a result of increased intraluminal pressure from fecal impaction leading to ischemia of the bowel.

Subsequently, ulceration may occur in perforation that can lead to severe hemodynamic compromise. Patients usually present with acute abdomen and are diagnosed by a CT scan of the abdomen and performing a colonoscopy. Diagnostic delay may lead to perforation and septic shock with a mortality rate as high as 60% [3, 4].

More than 200 cases of stercoral colitis have been published with a focus on the radiographic characteristics of stercoral colitis and management of abdominal perforation as a complication of colitis. However, only a few cases of ischemic colitis that are complicated by stercoral colitis have been published so far [5, 6]. Hence, the purpose of this case report is to describe the potential challenges in the diagnosis and management of stercoral colitis with ischemic colitis.

We followed CARE reporting guidelines in publishing our case report.

## Case presentation

An 87-year-old male presented to emergency department with complaints of severe abdominal pain and distention for 5 days. The pain was described as diffuse, non-radiating, moderate in intensity and associated with distention and bloating. Patient denied nausea, vomiting, fever, chills, bright blood per rectum and melena. There was no history of shortness of breath, palpitations, anorexia or weight loss. Patient has a past medical history significant for hypertension, hypercholesterolemia, benign colon polyps and chronic constipation, for which he was on bisacodyl tablets and polyethylene glycol at home. The last colonoscopy was performed 2 years ago, which showed diverticulosis and hyperplastic sigmoid colon polyp. There was no history of prior abdominal surgeries. Other home medications include oxycodone and tramadol for chronic back pain, statins, atenolol and bumetanide. The patient was hemodynamically stable. On abdominal examination, there was moderate diffuse tenderness, but no rigidity or guarding. Rectal examination revealed impacted stool. The remainder of the physical examination was unremarkable.

**Fig. 1:**
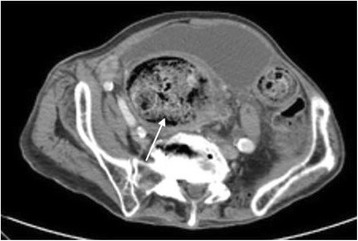
CT scan image of the abdomen showing large amount of retained stool in the colon with bowel wall thickening and fat stranding

Laboratory studies included WBC 15,700/mcL, hemoglobin 14.0 g/dL, and platelet count 214,000/mL. Basic metabolic panel was significant for anion gap metabolic acidosis [AG: 24] with lactate level of 6.2. Glucose, liver and renal function were normal. Urinalysis and chest x ray showed no evidence of infection. Blood cultures were obtained, which were subsequently negative. Abdominal x rays were obtained in the emergency department showing a large amount of gas and fecal retention throughout the colon and rectum with no evidence of free intraperitoneal air. CT scan of the abdomen and pelvis with contrast was performed and showed a large amount of retained stool throughout the rectum and colon with bowel wall thickening in the area, and infiltration of the pericolonic fat. CT scan was negative for intramural or extra-luminal air loculi, gas bubbles, or an abscess that might suggest a perforation process (Fig. 1).

**Fig. 2:**
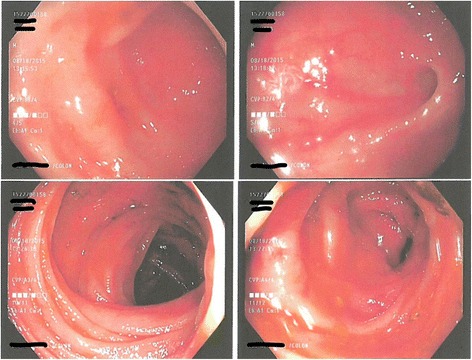
Colonoscopy image of descending colon showing edema, erythema of the colonic wall with sloughing of mucosa

**Fig. 3:**
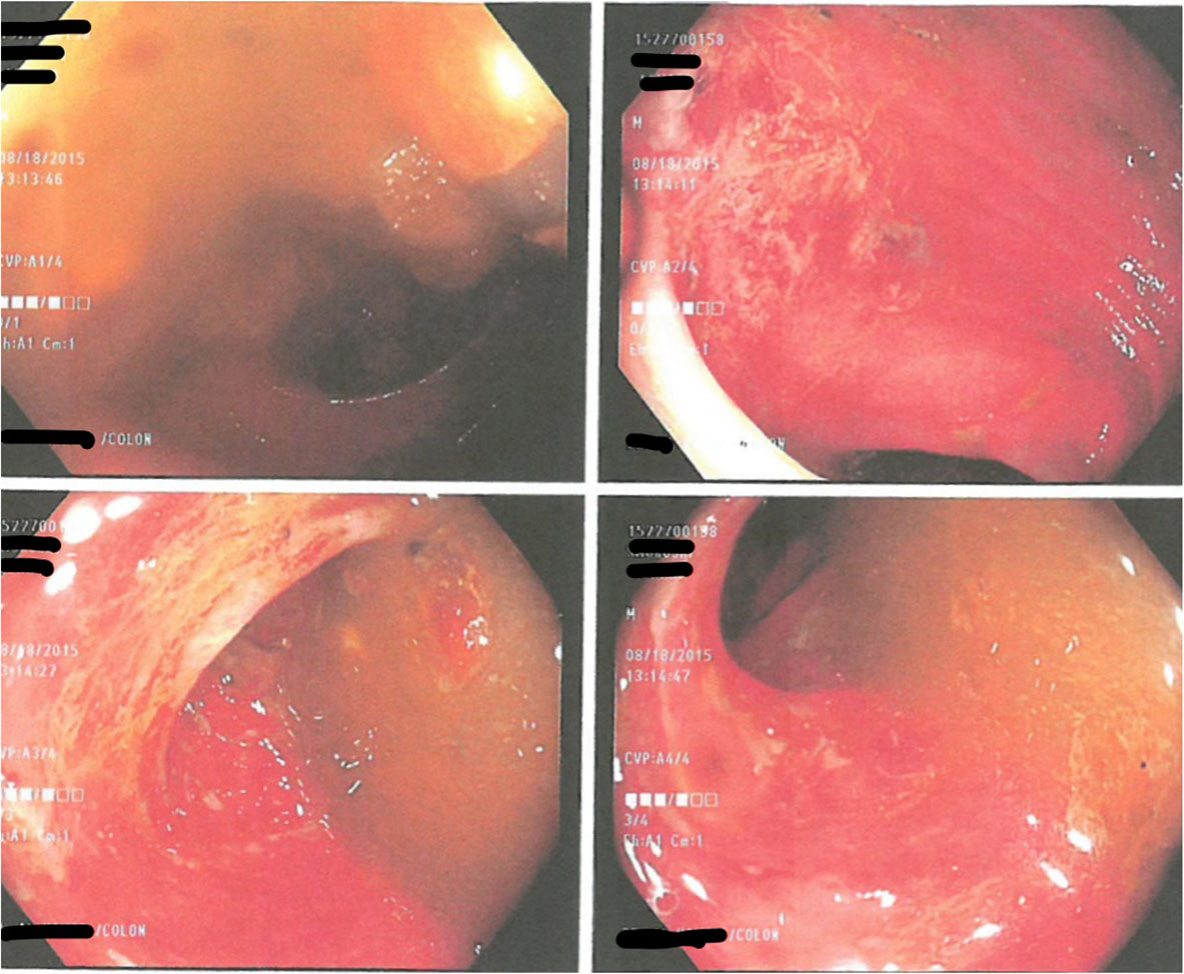
Colonoscopy image showing edema, erythema of the sigmoid colon wall with stercoral ulcer

In the emergency department, the patient was given milk of molasses enema and started on IV fluids, ciprofloxacin, and metronidazole. He was admitted in the inpatient unit and the gastroenterology team was consulted. The patient had several bowel moments afterwards. However, he still had a sensation of bloating and abdominal discomfort. White cell count and lactate trended down to 10,700/mcL and 4.3 respectively after fluid resuscitation and laxatives. Patient was taken to the endoscopic suit for colonoscopy after GoLYTELY bowel preparation. Colonoscopy showed a significant amount of solid stool in the rectal vault and left-sided colitis involving part of descending and recto-sigmoid colon with edema, erythema, and ulceration (Figs. 2 and 3). Manual disimpaction of stool was performed during colonoscopy. Multiple biopsies were obtained that were significant for focal active colitis with regenerative glandular changes and neural hyperplasia. After colonoscopy and manual disimpaction symptoms of bloating and abdominal discomfort resolved gradually and a repeat lactate came back within normal limits. The patient was discharged home in a stable condition.

## Discussion

Constipation and fecal impaction are common in elderly patients and can be secondary to colonic functional disorders, metabolic, neurologic disorders, pharmacotherapy (especially narcotics) and inappropriate diet and fluid intake. Stercoral colitis (SC) was first described in 1894 by Berry et al. and is a rare complication of chronic constipation and fecal impaction [7]. SC is defined as an inflammatory colitis that is caused by increased intraluminal pressure in the colon from the impacted feces and ischemic colitis (IC) is the condition that results when blood flow to the colon is reduced to a level insufficient to maintain cellular metabolic function.

Actual incidence and prevalence of the stercoral colitis and its complications are not known. The estimated post mortem incidence of stercoral ulcer ranges from 0.04% to 2.3%. Per the study published by Maurer et al., stercoral perforation of the colon was found in 0.5% of all surgical colorectal procedures, 1.2% of all emergency colorectal procedures, and 3.2% of all colonic perforations [8]. When stercoral colitis is associated with colonic perforation, a 35% mortality rate has been reported in the literature [9]. However, decreased morbidity and mortality have been observed in patients who were diagnosed earlier and managed appropriately.

To date, less than 200 cases of stercoral colitis have been reported in the literature. Intestinal perforation and solitary stercoral ulcers are commonly reported complications of stercoral colitis [10, 11]. Only 3 case reports and 2 case series of localized ischemic colitis with lactic acidosis are reported in total [12–14]. Extensive ischemic colitis involving right colon associated with stercoral colitis is only reported in one case report [15]. Our patient, presented with left sided ischemic colitis and stercoral ulcers (Table 1). It was unclear that the patient had ischemic colitis in advance before stercoral colitis or if the stercoral colitis resulted in colonic ischemia.

**Table 1:** Comparison of our patient with published data on stercoral colitis

Case #	Patient	Past Medical History	Presentation	Physical Examination Findings	Laboratory Findings	Imaging Findings	Course of Stay
**1 (our case)**	87-year-old male	HTN, Hypercholesterolemia, benign colon polyps, & chronic constipation	Severe diffuse abdominal pain with distension and bloating sensation of 5 days duration	**Abdominal exam**: moderate diffuse tenderness but no rigidity or guarding; **Rectal exam:** impacted stool	Leukocytosis and lactic acidosis	**AXR:** large amount of gas and fecal retention throughout the colon and rectum with no evidence of free intraperitoneal air; **Contrast-enhanced CT abdomen/pelvis:** large number of retained stools in the colon, bowel wall thickening and infiltration of peri-colonic fat	Findings suggestive of stercoral colitis complicated with ischemic colitis treated with I.V. fluids and antibiotics; Enema, followed by laxative and manual disimpaction of stools; symptoms were resolved and lactate levels returned to normal; patient became stable and discharged home
**2** ^**(12)**^	35-year-old male	Schizoaffective disorder	Diffuse cramping abdominal pain and constipation of 4 days duration	**Abdominal exam:** marked distention, diffuse tenderness to palpation, and stool palpable in the left lower quadrant with normal bowel sounds; **Rectal exam:** refused	Normal	**Contrast-enhanced CT abdomen/pelvis:** stool impaction with colonic wall thickening, but no small bowel obstruction, obstructing mass, or volvulus	Despite I.V. fluids and laxatives course was complicated with lactic acidosis and perforation of transverse colon with mucosal ulceration and focal ischemia. Patient underwent sub-total colectomy and was discharged with an ileostomy
**3** ^**(12)**^	26-year-old male	Long history of anxiety around using the restroom, after experiencing an earthquake while using the toilet at age 6	Constipation of 1 week; cramping abdominal pain in the lower quadrants and shortness of breath	**Abdominal exam:** distended and nontender, with stool palpable in the left lower quadrant and normal bowel sounds throughout; **Rectal exam:** hard stool palpated in the rectal vault	Normal	**AXR:** dilated colon with severe fecal impaction, without pneumoperitoneum; **Contrast-enhanced CT abdomen/pelvis:** fecal impaction with signs of bowel ischemia, but no free air or ascites were identified	Patient was treated with I.V. fluids, oral laxatives, and water enemas. Discharged home in stable condition
**4** ^**(13)**^ *limited data*	76-year-old male	DM, HTN, arrhythmia, chronic constipation	Acute abdomen; febrile	N/A	Leukocytosis	**CT abdomen/pelvis:** fecal impaction at recto-sigmoid colon; colon mucosal perfusion defect; pericolonic stranding; **Operation findings/Pathology:** Ischemic change from sigmoid to rectum with necrotic mucosa/Ischemia necrosis with mucosal sloughing	Alive; *limited information*
**5** ^**(13)**^ *limited data*	39-year-old male	ESRD, chronic constipation	Acute abdomen; hypotensive and febrile	N/A	Borderline leukocytosis	**CT abdomen/pelvis:** fecal impaction at recto-sigmoid colon with proximal dilatation; pericolonic stranding; **Operation findings/Pathology:** Ischemic patches over sigmoid colon with impending perforation/Ischemic and gangrenous change of the sigmoid colon	Dead, 3 days after CT; *limited information*
**6** ^**(13)**^ *Limited data*	83-year-old male	ARDS, HF, HTN, COPD, chronic constipation	Acute abdomen	N/A	Leukocytosis	**CT abdomen/pelvis:** fecal impaction at recto-sigmoid colon; colon wall thickening; colon mucosal perfusion defect; pericolonic stranding; **Operation findings/Pathology:** Ischemic change of small bowel and sigmoid colon/Transmural necrosis of sigmoid colon and mucosal necrosis of small bowel	Dead, 11 days after CT; *limited information*

*HTN* Hypertension, *AXR* Abdominal X-ray, *DM* Diabetes mellitus, *N/A* Not available, *ESRD* End-stage renal disease, *ARDS* Acute respiratory distress syndrome, *HF* Heart failure, *COPD* Chronic obstructive pulmonary disease

7

Common locations for stercoral colitis are anterior rectum, anti-mesenteric border of the recto sigmoid junction, and the apex of the sigmoid colon which are described as the “watershed” area of the colon. The recto-sigmoid junction has a relatively low blood supply from and often inefficient or absent anastomosis between the branch of the inferior mesenteric artery and the branch of the superior rectal artery, referred to as Sudeck’s point. Therefore, these patients are at risk of ischemia, particularly related to hypoperfusion. The mechanism by which fecal impaction and chronic constipation can lead to stercoral inflammation, ulceration, and subsequently perforation is proposed to be distention of the colonic lumen and increased pressure from dry desiccated fecal material (also called fecaloma) which, if left untreated may result in perforation and peritonitis [12].

Per published reports, the usual presentation of stercoral colitis complicated with ischemic colitis is the colicky abdominal pain, which may be diffuse or localized in a patient with a history of chronic constipation. Physical examination findings are consistent with tenderness on palpation with signs of focal or diffuse peritonitis. The CT scan findings of stercoral colitis include dilated sigmoid colon and/or rectum with subsequent thickening of the wall. This likely symbolizes edema caused by focal ischemia, necrosis and ulceration. Additionally, stranding of the peri-colonic fat in an area that shows fecal impaction suggests ischemic colitis or wall edema. While the presence of intramural or extra luminal air loculi, bubbles of gas, or an abscess, suggests colonic perforation. Our patient’s CT scan findings correlate with other case reports in regards to the presence of fecal impaction and bowel wall thickening suggestive of stercoral colitis [9, 10]. Endoscopic features of stercoral enterocolitis complicated with ischemic colitis include mucosal edema, erythema, ulceration and obstructing large fecolith at the angulation of sigmoid-descending colon junction as reported by Cohen et al. [16]. Our patient has similar findings on colonoscopy.

Once the diagnosis made, the patient should be managed appropriately and promptly to avoid morbidity and mortality from bowel perforation and peritonitis. Non-operative management with bowel regimen, enemas, manual fecal disimpaction via per rectal examination or endoscopy considered standard of care. However, endoscopic guided disimpaction is considered the standard of care [17, 18]. Operative management is reserved for patient presenting with signs of peritonitis secondary to colonic wall perforation and includes surgical resection of the dilated colon. Our patient was managed with early detection and treatment to avoid this catastrophic consequence. Hence, early diagnosis and treatment with bowel cleansing and fecal disimpaction are essential to avoid the fatal consequence of such condition.

## Conclusion

In summary, stercoral colitis complicated with ischemic colitis can lead to focal ulceration, perforation, peritonitis, septic shock and eventually death if left undiagnosed or if not treated. Familiarity with the CT scan and endoscopic findings is very important, as it enables physicians to make the diagnosis and start the appropriate treatment as fast as possible. The imaging findings that should be kept in mind whenever stercoral colitis is suspected includes the presence of fecal impaction, thickening of the colonic wall, stranding of the peri-colonic fat, and the presence of extra luminal gas bubbles or abscess suggesting stercoral perforation.

## Abbreviations

IC: Ischemic colitis
SC: Stercoral colitis
